# Comparison of the neuropoietic activity of gene-modified versus parental mesenchymal stromal cells and the identification of soluble and extracellular matrix-related neuropoietic mediators

**DOI:** 10.1186/scrt418

**Published:** 2014-02-26

**Authors:** Irina Aizman, Brenna J Tirumalashetty, Michael McGrogan, Casey C Case

**Affiliations:** 1 Department of Research, SanBio, Inc., 231 S. Whisman Rd, Mountain View, CA 94041, USA; 2 Department of Biology, San Francisco State University, National Science Foundation master’s fellowship DGE1011717, San Francisco, CA, USA; 3 Department of Production Development, SanBio, Inc., 231S. Whisman Rd, Mountain View, CA 94041, USA

## Abstract

**Introduction:**

Transplanting mesenchymal stromal cells (MSCs) or their derivatives into a neurodegenerative environment is believed to be beneficial because of the trophic support, migratory guidance, immunosuppression, and neurogenic stimuli they provide. SB623, a cell therapy for the treatment of chronic stroke, currently in a clinical trial, is derived from bone marrow MSCs by using transient transfection with a vector encoding the human Notch1 intracellular domain. This creates a new phenotype, which is effective in experimental stroke, exhibits immunosuppressive and angiogenic activity equal or superior to parental MSCs *in vitro*, and produces extracellular matrix (ECM) that is exceptionally supportive for neural cell growth. The neuropoietic activity of SB623 and parental MSCs has not been compared, and the SB623-derived neuropoietic mediators have not been identified.

**Methods:**

SB623 or parental MSCs were cocultured with rat embryonic brain cortex cells on cell-derived ECM in a previously characterized quantitative neuropoiesis assay. Changes in expression of rat neural differentiation markers were quantified by using rat-specific qRT-PCR. Human mediators were identified by using expression profiling, an enzymatic crosslinking activity, and functional interference studies by means of blocking antibodies, biologic inhibitors, and siRNA. Cocultures were immunolabeled for presynaptic vesicular transporters to assess neuronal specialization.

**Results:**

Among six MSC/SB623 pairs, SB623 induced expression of rat neural precursor, oligodendrocyte, and astrocyte markers on average 2.6 to 3 times stronger than did their parental MSCs. SB623 expressed significantly higher FGF2, FGF1, and BMP4, and lower FGFR1 and FGFR2 levels; and human FGF1, FGF2, BMPs, and HGF were implicated as neuropoietic mediators. Neural precursors grew faster on SB623- than on MSC-derived ECM. SB623 exhibited higher expression levels and crosslinking activity of tissue transglutaminase (TGM2). TGM2 silencing reduced neural precursor growth on SB623-ECM. SB623 also promoted the induction of GABA-ergic, but not glutamatergic, neurons more effectively than did MSCs.

**Conclusions:**

These data demonstrate that SB623 cells tend to support neural cell growth more effectively than their parental MSCs and identify both soluble and insoluble mediators responsible, at least in part, for enhanced neuropoietic potency of SB623. The neuropoiesis assay is a useful tool for identifying beneficial factors produced by MSCs and their derivatives.

## Introduction

After the acute stroke, the evolution of ischemic tissue and the restoration of neuronal functions continue for months and even years [1] and include the long-lasting enhancement of neurogenesis [2,3]. In contrast to normal adult neurogenesis that supplies neurons for the olfactory region and hippocampus, this injury-generated neurogenesis produces neurons that migrate toward damaged brain tissue [4,5]. The vast majority of these neurons, however, survive for only a few days [3], likely because of a hostile and deficient environment in the penumbra and the lack of connections. New cell therapeutic approaches, such as intracranial transplantation of mesenchymal stromal cells (MSCs), target the brain microenvironment by decreasing the inflammation, augmenting angiogenesis, promoting the formation and integration of newborn neurons, and supporting neural tissue, as well as by encouraging synaptic connection from damaged neurons (reviewed in [6,7]). These effects are thought to be mediated primarily via paracrine factors produced by the transplanted cells; however, the precise mechanisms are still unknown.

SB623 cells, an MSC derivative, are currently in clinical trial as an intracranial cell therapy for chronic stroke. They are derived from MSCs by using transient transfection with a vector encoding the human Notch1 intracellular domain (NICD1), followed by a 1-week selection and subsequent propagation. They demonstrate positive effects in ischemic stroke and Parkinson animal models [8,9]. This manufacturing process creates a cell population that appears superior to the parental MSC in benefits it provides to the degenerating neural tissue. In i*n vitro* studies comparing SB623 cells to their parental MSC, SB623 show equal or better immunosuppressive properties [10], improved angiogenic potency [11], more robust growth of neural cells on SB623-derived extracellular matrix (ECM) [12], and equal protection of neurons or hippocampal slices from oxygen-glucose deprivation [13].

The main focus of the present study is to test whether SB623 cells differ from the parental MSC population in their neuropoietic potency (that is, in their ability to stimulate the proliferation of neural precursors and their differentiation into neurons and glia.

We recently characterized a microplate assay for quantitative analysis of MSC-driven neuropoiesis [14]. The assay uses direct coculturing of primary embryonic rat neural cells with human MSCs on cell-derived ECM, which serves as a ”universal” substrate for the serum-free growth of cells with different attachment requirements. Within 1 week, MSCs cell number-dependently stimulate the growth of rat neural precursors, astrocytes, oligodendrocytes, and neurons; this stimulation can be quantified by measuring mRNA expression of corresponding rat markers directly from coculture lysates. The system enables a quantitative comparison of MSC lots or MSC derivatives, as well as studying of both diffusible and locally acting ECM-associated mediators of neural cell growth.

Recently, MSCs have been shown to promote synaptic transmission, both *in vivo* and *in vitro *[15,16]. In cultures of embryonic cortical cells, the differentiation of cortical neurons into glutamatergic and GABAergic is predetermined early, before neurons extend axonal processes (reviewed in [17]). Subsequent neuronal differentiation, cell-cell contact, and exposure to soluble factors [18,19] all lead to the localized induction of presynaptic varicosities representing complex assemblies of components of hemi-presynapses, including either vesicular glutamate (VGLUT) or vesicular GABA transporters (VGATs), which are responsible for the uptake of corresponding neurotransmitters into synaptic vesicles. These presynapses subsequently cluster postsynaptic hemisynapses in apposing dendrites and together undergo both spatial and temporal dynamic changes. Recently, MSCs were implicated in enhancing GABAergic transmission in cocultures [16]. Here we compared the effects of MSCs and SB623 by quantifying presynaptic puncta in cocultures.

The question of why SB623 cell-derived ECM is more neurosupportive is also addressed here. Tissue transglutaminase (TGM2) is a signature MSC protein [20], a multifunctional enzyme best known for its transamidating function, which causes the posttranslational modification of substrates by *de novo* formation of covalent bonds. TGM2 crosslinks itself to fibronectin and fibrinogen and generates highly stable covalent protein heterocomplexes in ECM (reviewed in [21]). This protein also has numerous nonenzymatic activities; in particular, it functions in ECM as an adaptor/scaffolding, thus promoting cell adhesion and migration. TGM2 was recently found to be differentially present in MSC- and SB623-ECM [22]. This finding, together with our prior observations that SB623-derived ECM is both more favorable for neural cell growth and less “fragile” during purification than MSC-derived ECM, prompted us to test the hypothesis that elevated TGM2 levels contribute to the superior neurosupportive properties of SB623-derived ECM.

Thus, the major goal of this study was to compare the neuropoietic activity of SB623 cells with that of their parental MSCs and identify some of the mediators of this effect. The comparison was conducted (a) in direct cocultures of either MSC or SB623 cells with rat neural cells, allowing the identification of soluble and cell-surface-associated human mediators, and (b) by culturing neural cells on MSC- or SB623-derived ECM, allowing the identification of insoluble, ECM-bound factors. These approaches, in combination with blocking/neutralization agents, were used to implicate specific human factors in the SB623-enhanced induction of neural precursors, astrocytes, and oligodendrocytes, whereas the effects on neuronal differentiation became evident through analysis of presynaptic formation.

## Materials and methods

### MSC and SB623 cell preparations and culturing

MSCs were prepared from bone marrow aspirates purchased from Lonza (Walkersville, MD, USA), which obtained all the necessary consent forms; MSC and SB623 cell preparation as well as their characterization was previously described [12]. MSCs and SB623 from eight donors were used in this study. All the donors were young men 19 to 25 years old; the panel used here included different races. For coculture experiments, cryopreserved cell aliquots were thawed, washed, and resuspended in a neural growth medium consisting of basal medium for embryonic neuronal cells (Neurobasal) supplemented with B27 and 0.5 m*M* L-alanyl-L-glutamine (GlutaMAX) (NB/B27/GLX) (all from Invitrogen).

For other uses, cells were plated at 3 to 5 × 10^4^/cm^2^ in α-minimum essential medium (Mediatech, Inc., Manassas, VA, USA) supplemented with fetal bovine serum (HyClone, Logan, UT, USA) and penicillin/streptomycin (Invitrogen) (αMEM/FBS/PS) and grown for various times. Specifically, for mRNA preparation, cells were grown for 5 days and then lysed; for measuring TGM2 activity in cell lysates, cells were grown for 5 days and serum-starved for an additional 2 days; for the preparation of conditioned medium, cells were grown for 3 to 4 days, and then the medium was replaced with NB for 1 hour and then again with fresh NB for 24 hours, followed by conditioned medium collection and storing at -80°C. Culturing for ECM preparation is described later.

### Plate coating

For the comparison of MSC and SB623 potency in the quantitative neuropoiesis coculture assay or for immunofluorescence, plates were coated with SB623 cell-derived ECM, as described previously [12]. In brief, SB623 cells were plated at 3 × 10^4^ cells/cm^2^ in 96-well plates (Corning Inc., Corning, NY, USA) or on glass coverslips (Fisher Scientific, Pittsburgh, PA, USA) placed into 12-well plates (Corning). Cells were grown for 5 days, and then the medium was changed to serum-free, and culturing continued for 2 days. For comparison of MSC- and SB623-derived ECM, or for studying TGM2 gene silencing, oxygen-rich surface (CellBIND) 96-well plates (Corning) were used instead of regular tissue culture-treated plates, and the cells were grown for 6 days followed by serum deprivation for 1 day. Cell removal and ECM purification were done as described previously [12]. In brief, cells were treated with 0.2% Triton X-100 (Sigma-Aldrich, St. Louis, MO, USA), then 0.33% NH_4_OH (Sigma-Aldrich), followed by soaking in PBS and storing at 4°C for no longer than 3 weeks.

Ornithine/fibronectin coating (Orn/Fn) was prepared as described in [14].

### Preparation of rat embryonic brain cortical cells

Rat embryonic day 18 (E18) brain cortex pairs were purchased from BrainBits (Springfield, IL, USA), and a cell suspension was prepared as described previously [14].

### ECM-based quantitative neuropoietic assay

All ingredients (medium, test substances, human and rat cells) were added to an intermediate uncoated 96-well plate first, 2.2 times final amounts per well, each condition in quadruplicates; the resulting suspensions were mixed. ECM-coated plates were brought to room temperature, and the PBS removed. Concentrations and cell densities reported later are indicated as final. Rat neural cells were plated at the constant density of 1.5 × 10^4^ cells/cm^2^ (5 × 10^3^ cells/well); human cells were used at varying densities. For the quantitative comparison of neuropoietic activities, human cells were plated at 125, 250, and 500 cells/well, resulting in human/rat cell ratios 1:40, 1:20, and 1:10. In the testing of inhibitory substances, the human/rat cell ratio was 1:25, or as indicated. Cell suspensions from the intermediate plates were distributed to duplicated ECM-coated culture plates at 100 μl/well. Cultures were grown for 5 or 7 days, and then culture medium was completely aspirated, and cells were lysed in 20 μl/well lysis and RNA stabilization buffer (SideStep; Agilent Technologies, Santa Clara, CA, USA) according to the manufacturer’s protocol.

### Gene expression quantification

To determine gene-expression levels in human cells, mRNA was purified by using RNA purification kit (RNeasy) and DNA- free RNA set, both from Qiagen (Germantown, MD, USA), according to the manufacturer’s instructions; and 5 ng of purified RNA was used per quantitative RT-PCR (qRT-PCR). One-step qRT-PCR was performed by using QuantiTect Probe RT-PCR Master Mix (Qiagen) and preoptimized Taqman assays (Applied Biosystems/Life Technologies), according to the manufacturer’s protocol. Expression levels were normalized to those of glyceraldehyde 3-phosphate dehydrogenase (GAP). For the quantification of neuropoietic effects exerted by human cells or ECM on rat neural cells, the expression of rat neural markers or human GAP was determined directly in SideStep lysates diluted in water 1:10, as described in [14].

The following Taqman expression assays were used: rat-specific: nestin (Rn00564394_m1), GFAP (Rn00566603_m1), and CNP (Rn01399463_m1); and human-specific: GAP (4333764 F), BMP4 (Hs00370078_m1), FGF2 (Hs00266645_m1), FGF1 (Hs00265254_m1), FGFR1 (Hs00915142_m1), FGFR2 (Hs01552926_m1), EGF (Hs01099999_m1), HB-EGF (Hs00181813_m1), HGF (Hs00300159_m1), and TGM2 (Hs00190278_m1).

### Growth factor neutralization and gene silencing experiments

Anti-FGF2 monoclonal antibodies, clone bFM1 (neutralizing) and clone bFM2 (nonneutralizing) were from Millipore (Billerica, MA, USA). Polyclonal goat anti-FGF1, normal goat IgG control (both used at 5 μg/ml), and recombinant human noggin were all from R&D Systems. Recombinant FGF1 and FGF2 (used at 5 ng/ml) were from Peprotech.

For silencing experiments, human cells were plated at 2 × 10^5^ cells/well in six-well plates in αMEM/FBS. On the next day, cells were transfected with siRNA, either ON-TARGETplusSMARTpool human HGF siRNA, TGM2 siRNA, or control nontargeting pool by using a transfection reagent (DharmaFECT1) (all reagents from Thermo Scientific Dharmacon, Lafayette, CO, USA) according to the manufacturer’s instructions. The next day, cells were harvested by trypsinization. The viability of all transfectants was >90%. Cells to be tested in cocultures were then washed once with αMEM/FBS and twice with NB. Cells to be used for ECM production were plated into CellBind 96-well plates and grown for 6 days, and then serum-starved for 1 day; and the ECM was purified.

Aliquots of all transfectants were also plated in αMEM/FBS/PS to test the silencing efficiency on day 6 by using qRT-PCR, as well as for controlling actual cell numbers by using lactate dehydrogenase (LDH) assay in cell lysates.

### LDH activity assay

For cell number normalization, LDH activity in cell lysates was determined as described in [12] by using the Cytotoxicity Detection Kit (Clontech Laboratories, Mountain View, CA, USA).

### Immunofluorescence and quantification of hemi-presynapses

The detection of rat nestin, GFAP, and CNP was described previously [14]. For the detection of vesicular glutamate- and GABA-transporters (VGLUT and VGAT), cells were plated on Orn/Fn-coated glass coverslips, cultured for 7 and 11 days, respectively, and fixed with 4% paraformaldehyde/5% sucrose. The coverslips were then blocked with 3% bovine serum albumin/0.3% TritonX-100 (both from Sigma-Aldrich) for 1 hour and incubated with rabbit anti-VGAT or guinea pig anti-VGLUT (both from Synaptic Systems, Goettingen, Germany) for 1 hour. After washing, the coverslips were incubated for 1 hour with donkey DyLight 549-conjugated AffiniPure anti-rabbit F(ab’)_2_ IgG fragments and Alexa Fluor 488-conjugated AffiniPure anti-Guinea Pig F(ab’)_2_ IgG fragments (Jackson Immunoresearch). After washing, the slips were mounted with ProLong Gold antifade reagent containing 4′,6-diamidino-2-phenylindole (DAPI) (Invitrogen). Fluorescence microscopy was done by using Nikon Eclipse50i microscope (Nikon Instruments, Melville, NY, USA) and a Nikon Digital Camera, DXM1200C.

For the quantification of immunostaining per positive cell, neural cells were cultured either alone or with MSCs or SB623 at human/rat cell ratio of 1:20 for either 5 days (Nestin/DAPI and GFAP/DAPI staining) or 12 days (CNP/DAPI staining). Images of duplicated cultures, two images per culture (total, four fields), were acquired by using 100× magnification; and neuromarker signals were quantified by using ImageJ. The numbers of positively stained cells were determined manually by overlaying an image of immunostained culture with a corresponding DAPI image. The results were expressed as staining per cell. For the quantification of VGLUT- and VGAT-puncta*,* duplicated cultures were set for each condition*.* Microphotographs of 10 fields (five fields per culture), which included easily traceable neurites with punctated staining, were taken at 400× magnification by using the same exposure time for each antigen. Maximum contrasted images were printed at full page on a Bizhub 280 (Konica Minolta) printer, and the numbers of puncta were counted per neurite length between the first and last visible puncta on the image.

To prevent sampling bias, printed images were placed at random order, and counting was done on unidentified images. One to six neurite segments were counted per image, and the numbers of puncta/100 μm were averaged. The identity of printed images was then determined by using the original digital image.

### TGM2 activity assay

A 96-well plate was coated with poly-L-lysine (PLL; Sigma-Aldrich) at 10 μg/ml in PBS overnight at 4°C, and then rinsed once with PBS and once with water. Monolayers of MSCs or SB623 were rinsed in PBS and then lysed in a mammalian cell lysis/extraction reagent (CelLytic M, Sigma-Aldrich) for 15 minutes at room temperature, with shaking. Lysates were centrifuged at 13,000 rpm for 15 minutes at 4°C, and supernatants immediately analyzed or stored at -80°C. Cell lysate (10 μl) was added to 90 μl TGM2 activity assay buffer consisting of 1% Triton-X100, 50 m*M* Tris–HCl (pH 7.4), 150 m*M* NaCl, 1 m*M* DTT, and 5 m*M* CaCl_2_, followed by the addition of 20 μl 2.5 m*M* biotinylated cadaverine (B-Cad, Sigma-Aldrich). The plate was incubated at room temperature with gentle shaking for 30 minutes and rinsed twice for 5 minutes in 50 m*M* Tris, 3 m*M* EDTA. The plate was then blocked for 30 minutes with 3% BSA/50 m*M* Tris; and horseradish peroxidase-streptavidin conjugate (1:5,000, Jackson Immunoresearch) was added for 30 minutes. After three washes, 3,3′,5,5′-tetramethylbenzidine (1 × TMB Substrate Solution; eBioscience) was added, and absorbance was measured at 650 nm against a standard curve, which was based on dilutions of peroxidase-streptavidin conjugate. TGM2 activity was normalized to total protein content in lysates, which was determined by using a MicroBCA protein assay kit (ThermoScientific, Rockford, IL, USA).

### TGM2 Western blotting

MSCs and SB623 cells were grown in six-well plates, and ECM prepared as described earlier. ECMs were scraped and solubilized in 100 μl/well of Tris-Glycine SDS sample buffer (Life Technologies), and then boiled at 95°C for 5 minutes and centrifuged at 13,000 rpm for 15 minutes. Sample aliquots were electrophoresed on two identical 4% to 20% Tris-Glycine gels (Life Technologies); one was then transferred to nitrocellulose, and another one stained with a Coomassie G-250-based solution (SimplyBlue SafeStain, Invitrogen). The blot was blocked in 5% milk in Tris-buffered saline with 1× Tween-20 for 2 hours at room temperature and incubated for 2 hours with human-crossreactive mouse anti-guinea pig TGM2 monoclonal antibody (LifeSpan Biosciences). After three washes, the blot was incubated with peroxidase-conjugated donkey anti-mouse IgG antibody (Jackson Immunoresearch). TGM2 was visualized by using SuperSignal WestPico (Thermo Scientific). Densitometry was performed on the blot and on the stained gel by using Alpha Innotech imager and ImageJ software. The TGM2 signal was normalized to the total protein in each sample.

### Statistics

Error bars indicate standard deviation, unless indicated otherwise. Paired *t* test was used for calculating *P* values. A *P* value of less than 0.05 was considered to be significant.

## Results

### Comparison of neuropoietic activity of SB623 and MSCs

To observe differences in the neuropoietic potency of MSCs versus SB623 by using immunostaining, cocultures with rat embryonic cortex cells (human/rat cell ratio 1:10) were grown on ECM-coated glass and stained for rat neural markers. Time points were chosen based on our previous study (Aizman *et al*. 2013), which characterized the growth and differentiation of neural cells/MSCs cocultures. At these specific time points, the differences between neural cells alone and cocultures were large, and the protein expression of rat neural markers was directly dependent on the numbers of MSCs and SB623 cells added.

**Figure 1:**
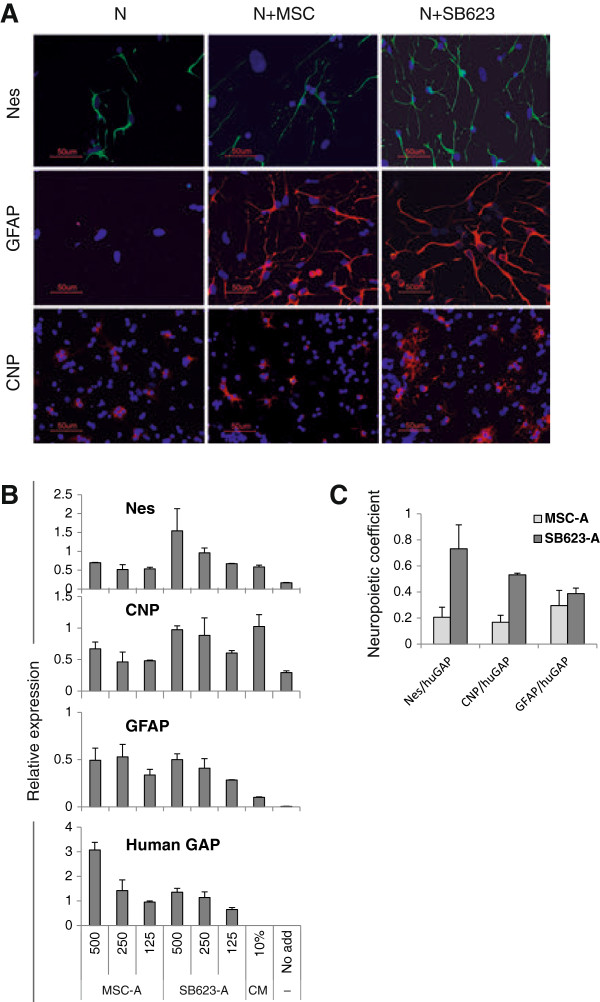
**Comparison of neuropoietic activity of SB623 and MSCs in cocultures with rat embryonic neural cells. (A)** Rat neural cells were grown in the presence or absence of MSCs or SB623 (rat/human cell ratio was 10:1) and immunostained for rat nestin (upper panel) and GFAP (middle panel) on day 5, or for CNP on day 12. Nuclei were stained with DAPI **(B)** An example of microplate neuropoiesis assay data: a comparison of rat neural differentiation marker induction in cocultures of rat cells with MSCs and SB623 from Donor A. Rat neural cells (5,000/well) were cocultured with 500, 250, and 125 cells/well of either MSCs or SB623; and expression of rat-specific nestin, GFAP, and CNP, and human-specific GAP was quantified by using qRT-PCR. Stimulation with 10% MSC-CM and no stimulation (“No add”) were used as positive control and background, respectively. Relative units correspond to standard samples used in qPCR run. Error bars represent SD of biologic duplicates. **(C)** Neuropoietic coefficients of MSCs and SB623 from Donor A were calculated based on data presented in **(B)** by first subtracting the background expression of a corresponding neural marker and then normalizing the expression of the neural marker to the human GAP for each number of human cells per well, followed by averaging normalized values. Error bars represent SD from three normalized values.

Staining for rat nestin, a marker of neural precursors and stem cells, on day 5 (Figure 1A, upper row of images) demonstrated that in cocultures with SB623, nestin-positive (Nes^+^) cells were present in higher quantities and were brighter, compared with MSC cocultures, whereas in the absence of human cells, only a few Nes^+^ cells were seen, with less-developed processes. Staining for GFAP, an astrocyte marker, on day 5 (Figure 1A, middle row) revealed well-developed astrocytes present in both SB623 and MSC cocultures, whereas no staining was observed in the absence of human cells. Immunostaining for an oligodendrocyte marker, CNP, on day 12 (Figure 1A, lower row) revealed extensive cytoplasmatic CNP staining in cocultures with SB623 cells, limited perinuclear staining in cocultures with MSCs, and barely detectable staining in the absence of human cells. Cocultures were also stained for the neuronal marker MAP2, and no difference between MSC- and SB623-cocultures was detectable on days 5 and 9 in either the numbers of mature neurons or their dendrite outgrowth (see Additional file 1: Figure S1).

To compare MSC with SB623 cocultures quantitatively, we cocultured rat neural cells with three plating densities of human cells (125, 250, and 500 cells/well) and no human cells, and performed qRT-PCR for rat neural markers and human GAP (Figure 1B). Nestin and CNP, but not GFAP, expression levels were higher in SB623 cocultures, whereas human GAP levels were slightly higher in MSC cocultures. To analyze the data further, levels of neural marker expression in the absence of human cells were subtracted, and the resulting levels of each neural marker were normalized to corresponding human GAP for each number of human cells per well. The three normalized values were then averaged for either MSCs or SB623 cocultures and referred to as “neuropoietic coefficients” (Figure 1C). According to Figure 1C, for example, Nes neuropoietic coefficient of SB623 from Donor A (SB623-A) was 3.6 times higher than that of MSC-A. Table 1 demonstrates ratios of SB623- to MSC-neuropoietic coefficients for six donor pairs. SB623 cell preparations induced the three differentiation neuromarkers to a greater or similar, but not lower, extent than did MSCs. The averages of all three neuropoietic coefficients were found to be higher in the group of six SB623s compared with the group of six MSCs. Thus, overall, SB623 cells exhibited increased neuropoietic coefficients, compared with their parental MSCs, with some variability between donor pairs.

**Table 1:** SB623-to-parental MSC ratios of neuropoietic coefficients

	**Nes**	**CNP**	**GFAP**
Donor A	3.6	3.2	1.3
Donor B	3.9	3.5	7.3
Donor C	1.1	2.6	1.1
Donor D	1.4	2.9	1.9
Donor E	5.5	2.1	1.8
Donor F	2.3	1.8	2
**Average**	**3**	**2.7**	**2.6**
**SD**	**1.68**	**0.65**	**2.35**

Neuropoietic coefficients characterize the ability of MSCs or SB623 to induce a corresponding rat neural marker in defined conditions of the microplate neuropoiesis coculture assay. Pairs of SB623 and parental MSCs from six donors were compared in the assay; and neuropoietic coefficients for nestin, GFAP, and CNP were calculated, as explained in Figure 1, based on qRT-PCR data. The table represents ratios of SB623 to MSC coefficients for six donors; average ratios and standard deviations (SD) are in bold.

To support the qRT-PCR data, nestin, GFAP, and CNP expression also was quantified by using immunostaining. Image analyses were performed for cocultures with MSCs and SB623 of one donor (represented in Table 1 as Donor D) by measuring the total neuromarker staining in a field and dividing the value by the number of positive cells in the field (see Additional file 2: Figure S2). This method detected significantly stronger GFAP and CNP staining in cocultures with SB623 compared with the staining in cocultures with MSCs, whereas nestin staining trended higher (without reaching statistical significance) in SB623 cocultures compared with MSC cocultures, and was significantly higher in SB623 cocultures compared with neural cells alone.

### Expression of neuropoietic growth factors in SB623/MSC pairs

In preliminary experiments, we first selected neuropoietic factors of interest. Factors were chosen based on their expression in MSCs (based on prior publications) and on the effectiveness of their recombinant forms in inducing a neuropoietic response in our culture system. Then we compared MSCs and SB623 cells for the mRNA expression of the most potent of these factors: FGF2, FGF1, EGF, HGF, HB-EGF, BMP4, BMP2, and BMP6. We also tested the expression of two FGF receptors predominantly expressed by MSCs, FGFR1 and FGFR2. Table 2 summarizes data from eight SB623/MSC pairs (provided in Additional file 3: Table S1) by presenting the averaged ratios of SB623-to-MSC expression levels. The data show that FGF2, FGF1, and BMP4 levels increased, whereas FGFR2 strongly and FGFR1 mildly decreased in SB623 compared with MSCs. These differences were statistically significant. An FGF2 ELISA of conditioned medium detected higher FGF2 levels in SB623 compared with MSCs in four of six donors (donors C, D, E, and F; see Additional file 4: Figure S3). EGF levels were higher in five and lower in two of the eight SB623 lots compared with their MSCs. HGF expression exhibited great variability among donors (values differed up to 30-fold) and between pair members (in SB623 cells, it was either up or down, compared with MSCs).

**Table 2:** SB623-to-parental MSC ratios of mRNA expression levels

	**FGF1**	**FGF2**	**FGFR1**	**FGFR2**	**BMP2**	**BMP4**	**BMP6**	**HB-EGF**	**EGF**	**HGF**	**GAP**
SB623/MSC expression	1.3^a^	1.7^a^	0.7^a^	0.2^a^	ND	3.6^a^	1.6	0.9	1.5	0.7	
SD	0.27	0.47	0.13	0.08	ND	2.27	1.19	0.47	0.75	0.72	
Cp	28	26.5	26	27	35	30	33	29	30.5	31	21

Values represent averaged ratios obtained from qRT-PCR data on eight pairs of MSCs and SB623 for all factors, except for BMP2 and BMP6, where data from six MSC/SB623 pairs were used. SD, standard deviation; Cp, crossing point (threshold PCR cycle at which the signal becomes measurable), ^a^*P* < 0.05.

### Effects of neutralization and silencing of human growth factors on rat neural cell growth

**Figure 2:**
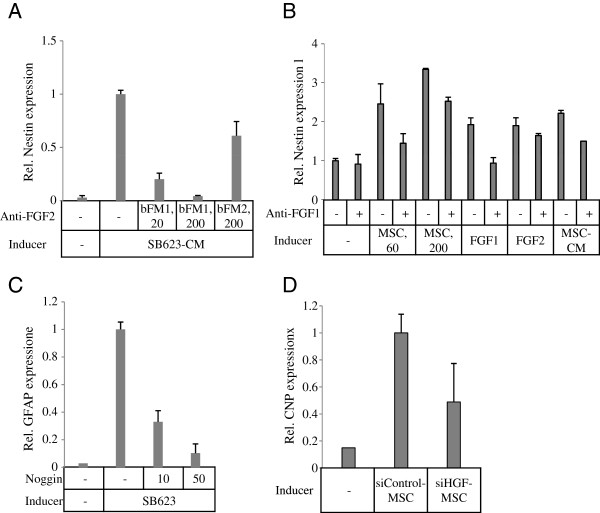
**Role of human growth factors in induction of rat neural markers in various culture settings; qRT-PCR. (A)** Effect of FGF2 inhibition on rat nestin. Rat neural cells were stimulated with SB623 cell-derived conditioned medium (25%), which increased the nestin expression. The presence of FGF2-neutralizing antibody (bFM1), but not FGF2-specific nonneutralizing antibody (bMF2), concentration-dependently inhibited the nestin increase. **(B)** Effect of human FGF1 inhibition on rat nestin. Rat neural cells were stimulated by MSCs (60 or 200 cells/well), MSC-CM (5%), or recombinant FGF1, or FGF2 (both at 5 ng/ml), which led to the induction of rat nestin. The application of the anti-human FGF1 neutralizing antibody decreased nestin mRNA levels induced by all stimuli but the recombinant FGF2. **(C)** Effect of BMP inhibition on rat GFAP induction. Rat GFAP was induced by coculturing rat neural cells with SB623 (200 cells/well). A BMP inhibitor noggin concentration-dependently decreased the GFAP induction. **(D)** Effect of HGF silencing in MSC on rat CNP induction. Rat CNP expression was stimulated by coculturing rat neural cells with MSC transfected with either HGF siRNA (siHGF) or control siRNA (siControl). A lower CNP induction was observed in cocultures with siHGF-transfectants.

Previously, we demonstrated that FGF2 is the major mediator of MSC- and MSC-CM-driven rat nestin induction in rat cortical cells/MSC cocultures [14]. Here we confirmed these results for SB623 cells: indeed, the anti-FGF2 blocking (bFM1), but not nonblocking (bFM2) monoclonal antibody, completely abrogated SB623-CM-driven nestin increase at 200 ng/ml and partially at 20 ng/ml (CM was used at 25%) (Figure 2A).

We also tested the contribution of FGF1 to the nestin increase in MSC cocultures (Figure 2B). FGF1-blocking antibody at 5 μg/ml completely abrogated the nestin increase driven by 5 ng/ml of recombinant FGF1, but not of FGF2. At the same concentration, the antibody only partially decreased the inductive effects of MSCs and MSC-CM. This suggested that human FGF1 was a contributing stimulus to the MSC- and SB623-driven nestin increase.

Rat GFAP expression in SB623 cocultures was reduced in a concentration-dependent manner by the addition of noggin protein, an antagonist of several BMPs, including BMP2 and BMP4 [23]. This indicated that SB623-driven rat GFAP expression in cocultures was mediated mainly by human BMPs (Figure 2C).

Although HGF expression was not consistently changed in SB623 cells compared with parental MSCs, it greatly varied between different cell lots. We decided to test human HGF contribution to the promotion of neurogenesis in cocultures, with the thought that if the contribution is substantial, then selecting MSCs with high HGF expression levels and using them for SB623 production might be beneficial. Human HGF was efficiently silenced by using siRNA: a very low residual HGF expression was found on day 6 after transfection. The next day after the transfection with either HGF siRNA (siHGF) or control RNA (siControl), transfected cells were used in cocultures with rat neural cells. After 5 days of coculturing, the expression levels of rat neural markers and human GAP were determined. Both Nes and GFAP levels were similar between siHGF and siControl cocultures, whereas CNP levels were reduced by about 75% to 85%. However, we noticed that the HGF silencing typically also reduced total human GAP expression by 25% to 50%, likely reflecting the reduced proliferation of siHGF transfectants. After normalization to huGAP, the relative expression levels of rat CNP showed a decrease (Figure 2D), and Nes and GFAP levels showed some increase (not shown) in cocultures with siHGF transfectants, compared with corresponding expression levels in cocultures with siControl transfectants. The silencing was performed on both MSCs and SB623, with very similar results. Furthermore, transfection of MSCs with either control siRNA or HGF siRNA resulted in a 20% to 30% decrease in the numbers of cells harvested after 6-day culturing, as compared with nontransfected cells. The number of harvested HGF transfectants was slightly smaller than that of the transfected control (see Additional file 5: Figure S4A). All three groups showed approximately 97% viability at harvesting. These results indicated that the HGF silencing likely slowed the cell proliferation more than the control transfection, which could explain the reduction observed in the total GAP expression in siHGF transfectants, as compared with the control.

We also tested siHGF and siControl transfectants for mRNA expression levels of a set of genes encoding growth factors (HGF, FGF2, HB-EGF, BMP6), ECM protein (LAMA4), enzyme TGM2, and another housekeeping protein (GDI), at two time points (Additional file 5: Figure S4B). Although HGF expression was strongly decreased in siHGF compared with siControl transfectants at both time points, none of the other tested genes showed downregulation. This confirmed that HGF silencing was specific. We concluded that human HGF was partly responsible for the induction of CNP, but not Nes or GFAP, in the cocultures.

### Effects of SB623 and MSCs on development of hemisynaptic puncta in neurons

**Figure 3:**
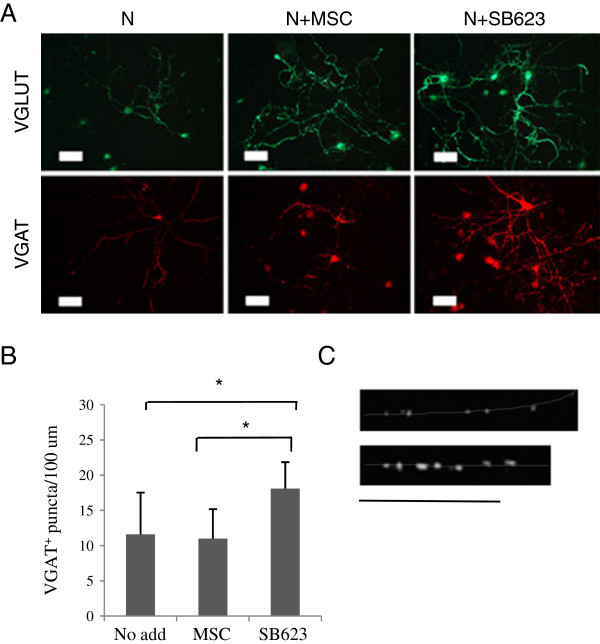
**Comparison of presynaptic puncta formation in cocultures of rat embryonic neural cells with MSCs or SB623; immunostaining. (A)** Immunostaining for VGLUT (day 7) and VGAT (day 11); rat/human cell ratio, 50:1. Bar, 50 μm. **(B)** Quantification of VGAT-immunoreactive puncta per neurite length (averaged from 10 microscopic fields, one to four neurites/field), day 11. **(C)** Typical distribution and size of VGAT-positive puncta in axonal processes in cocultures with MSCs (upper) or SB623 (lower), day 13. Neurites are outlined manually. Bar, 50 um.

We previously observed an approximately twofold increase of two rat neuronal markers: microtubule-associated protein 2 and Doublecortin, in cocultures with MSCs [14]. This was not, however, a sufficiently robust increase to permit the comparison of MSCs with SB623 (Additional file 1: Figure S1). Therefore, to compare the neuropoietic activity of MSCs and SB623 toward the neuronal lineage, we decided to use a more advanced differentiation characteristic, the development of presynaptic puncta, which were detected by the immunostaining of vesicle glutamate transporter (VGAT) and vesicle GABA transporter (VGAT), and by their distribution along neurites. The presence of either MSC or SB623 expedited the appearance of punctated VGLUT staining (most of the neurons stained positively) and, a few days later, punctated VGAT staining (Figure 3A). To compare quantitatively the effects of MSCs with those of SB623, we assessed the distribution of presynaptic puncta along neurites. No significant difference in the density of VGLUT puncta per neurite length was found in MSC- and SB623-cocultures (compared on day 8, not shown). However, we observed a significant increase (about 30%) in numbers of VGAT puncta per neurite length in SB623 cocultures, compared with MSC cocultures or with neural cells alone (Figure 3B). As shown on Figure 3C, in SB623 cocultures, VGAT puncta were also brighter and more regularly spaced than in MSC cocultures.

### Nes^+^-cell growth on MSC- and SB623-derived ECM

**Figure 4:**
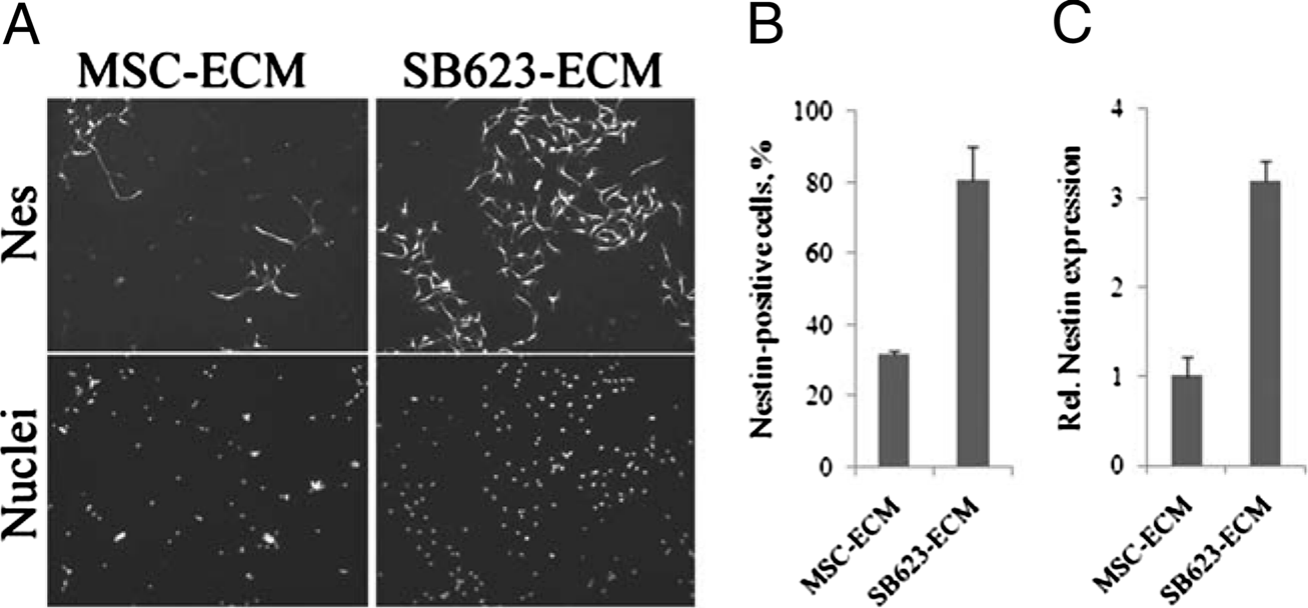
**Comparison of SB623- and MSC-derived ECM in supporting nestin-positive cell growth. (A)** Rat neural cells were grown for 5 days on MSC- or SB623-derived ECM and then immunostained for nestin (upper panel) and counterstained for nuclei (lower panel); magnification 100×. **(B)** Nestin-positive cells grown and stained as described in **(A)** were counted, and their numbers expressed as percentage of total nuclei. **(C)** Rat nestin expression in cells growing on MSC- or SB623-ECM was quantified by using qRT-PCR and normalized by the LDH activity released from either MSC- or SB623-ECM-producing cells during ECM preparation, correspondingly, to account for possible differences in cell numbers.

We previously demonstrated that ECM derived from SB623 supports a more robust growth of neural cells, as measured by the LDH activity in cell lysates after 2- to 3-week culturing [12]. Here we produced ECM from pairs of MSCs and SB623 and plated rat neural cells on it for the detection of nestin protein and mRNA. More Nes^+^-cells appeared on SB623-, than on MSC-derived ECM (Figure 4A), and counting showed a higher percentage of Nes^+^-cells on the SB623-ECM than on the MSC-ECM (Figure 4B). *Nes* gene expression was also quantified and normalized to the LDH activity released from MSCs and SB623 cells (during the lysis of cell monolayers for ECM preparation) to account for possible differences in the number of ECM-producing cells (Figure 4C). Thus, the quantification of both nestin immunostaining and mRNA expression showed that substantially more Nes^+^-cells were growing on SB623-ECM than on MSC-ECM.

### TGM2 in MSC and SB623; TGM2 silencing in ECM-producing cells reduces the ability of ECM to support Nes^+^ cell growth

**Figure 5:**
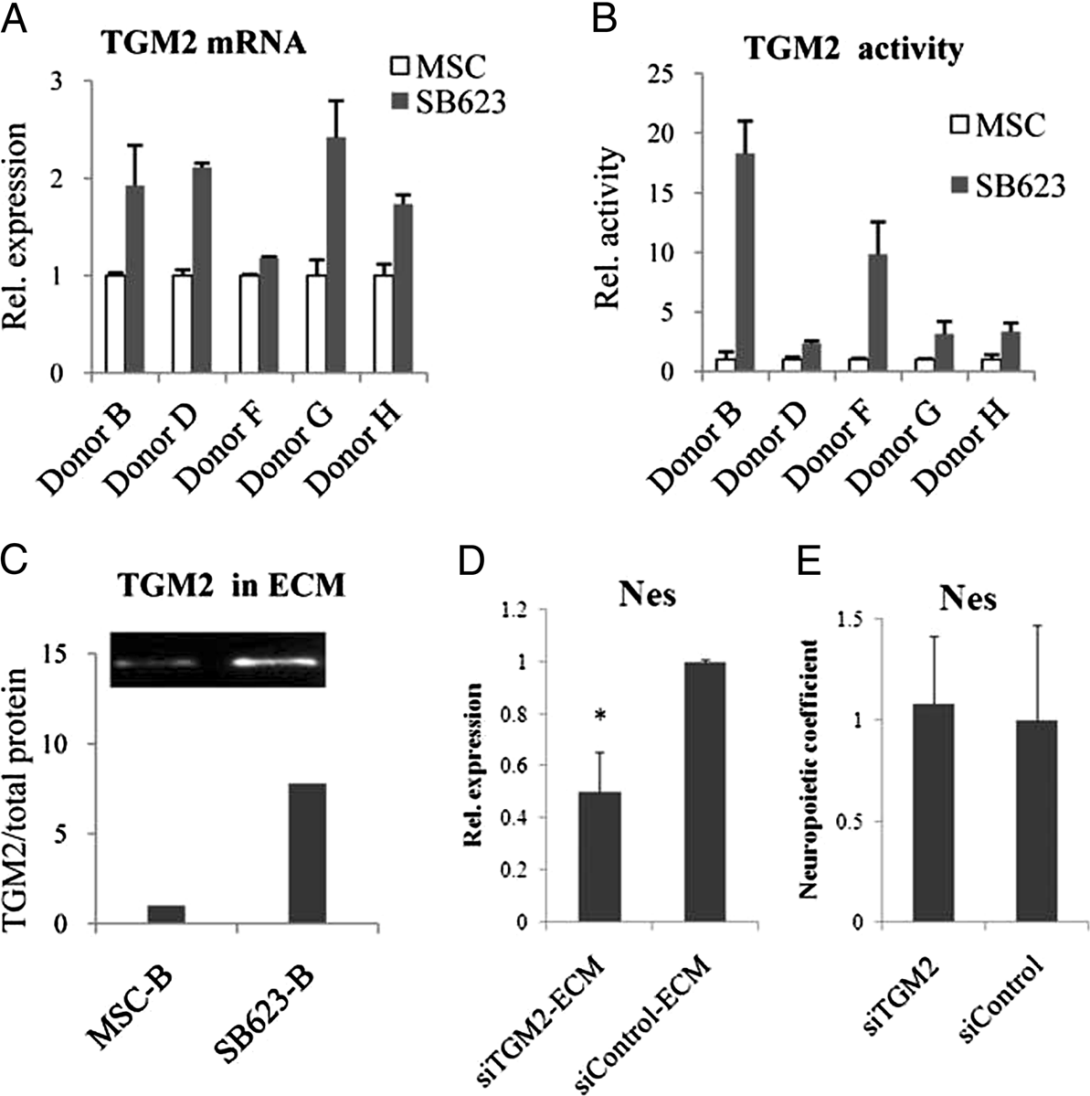
**Comparison of expression and activity of TGM2 in SB623 and MSC; its functional analysis in ECM using siRNA. (A)** Expression levels of TGM2 normalized to GAP were determined by using qRT-PCR in SB623/MSC pairs from several donors. Levels in SB623 cells were expressed relative to levels in parental MSCs, which were set on 1. **(B)** TGM2-crosslinking activity was measured by amounts of biotinylated cadaverine incorporated into PLL in the presence of SB623 or MSC cell lysates. The activity was then normalized to the total protein, and expressed compared with the parental MSCs, where the values were set on 1. **(C)** TGM2 was detected in ECM of MSCs and SB623 by using immunoblotting. The TGM2 signal was quantified densitometrically and normalized to the total ECM protein per lane. The total ECM protein was assessed by using duplicated gel: the gel was stained for protein; photographed; and the density of corresponding lane minus background determined. (The whole blot and gel are shown in Additional file 6: Figure S5). **(D)** Nestin expression was quantified by using qRT-PCR in rat neural cells grown on ECM produced by SB623 transfected with either siTGM2 or siControl. Nestin levels on siControl-ECM were set on 1. The graph represents means from three experiments; error bar represents standard error of mean; *P < 0.05. **(E)** Rat nestin mRNA expression in cocultures with either siTGM2 or siControl transfectants. The graph represents mean from two experiments.

A proteomic analysis of MSC- and SB623-ECM by using mass spectrometry [22] identified TGM2, an ECM cross-linking enzyme, as present at higher levels in SB623-ECM. Expression analysis confirmed systematically higher levels of TGM2 mRNA in SB623 than in MSC (in four of five donor-cell pairs) (Figure 5A). Next, we conducted a TGM2 enzymatic activity assay measuring the crosslinking of biotinylated cadaverine to PLL-coated microplate in the presence of total MSC or SB623 cell lysates. The activity was normalized to the total cell protein. The assay was TGM2-specific, because a TGM2-blocking antibody significantly reduced the incorporation of biotinylated cadaverine (not shown). The enzymatic activity assay demonstrated a higher crosslinking activity of SB623- than of MSC-lysates (Figure 5B). With immunoblotting, we also confirmed that in the majority of donor cell pairs tested, more TGM2 was found in SB623-ECM than in MSC-ECM (Figure 5C, Additional file 6: Figure S5).

To clarify whether ECM-bound TGM2 affects neural cell growth, SB623 cells were transfected with either TGM2-siRNA (siTGM2) or control siRNA, replated on the next day, and cultured for 7 days. The transfection with TGM2-siRNA effectively and specifically silenced the expression of TGM2: on day 7, residual TGM2 expression was around 5% of that of the control (not shown). After 7 days, one plate was used to purify ECM, and a duplicate plate was used in an LDH assay to generate data for the normalization to the number of ECM-producing cells. The ECM produced by siTGM2-transfectants, but not by siControl transfectants, was significantly more fragile (easily detachable) and required extreme care. We were unable to purify siTGM2-ECM from two of five SB623 lots, because siTGM2-ECMs were washed off. Successfully purified siTGM2- and siControl-ECMs (fully intact under the microscope) were used as substrates for rat embryonic cortical cells. After 5 to 7 days, rat nestin mRNA was measured and normalized to the LDH from ECM-producing cells. The normalized rat nestin mRNA expression was lower in cultures grown on siTGM2-ECM than in those grown on siControl-ECM (Figure 5D), indicating that the ECM produced by cells with silenced TGM2 had a decreased ability to support the growth of neural precursors.

We also tested the ability of siTGM2- and siControl transfectants to stimulate neuropoiesis in cocultures with rat neural cells (human/rat cell ratio was up to 1:10), and no difference in Nes levels was found (Figure 5E). This observation indicated that TGM2 silencing did not impact the ability of cells to mediate trophic support for Nes^+^-cells and also suggested that the transfectants’ health was comparable to that of the control. Indeed, although compared with the siControl transfectants, siTGM2 transfectants exhibited a slightly reduced cell proliferation (which is in agreement with a previous report [24]), no effect on the cell viability was found (Additional file 7: Figure S6A). When mRNA levels in siTGM2 were tested at days 2 and 6 after transfection and compared with corresponding levels in siControl cells, TGM2-siRNA transfectants showed substantial decreases in the level of TGM2, but did not show a decreased gene expression in a panel of other tested genes encoding growth factors HGF, FGF2, HB-EGF, and BMP6, ECM protein LAMA4, and the housekeeping protein GDI (Additional file 7: Figure S6B). This confirmed that the silencing of TGM2 was specific.

## Discussion

Here we present *in vitro* data indicating that SB623 cells, MSC derivatives, which are in development as a therapy for intracranial transplantations in the treatment of chronic stroke, more effectively stimulate neuropoietic responses and presynapse formation in neural cells than do their parental MSCs. We identified several factors that contribute to this difference.

To compare quantitatively the neuropoietic potency of SB623 cells with that of their parental MSCs, we adapted a previously characterized microplate neuropoiesis assay [14]. When rat neural cells are cocultured with human MSCs or SB623 cells at cell-plating densities within a previously determined linear range of the assay, rat neural cell proliferation and differentiation depend on the number of human cells. Therefore, at these conditions, ratios of rat neuromarker expression levels to human housekeeping gene-expression levels are constant and reflect the intrinsic potency of human cells to stimulate the rat neuromarker. These ratios, averaged from cocultures with several human cell-plating densities, are referred to as neuropoietic coefficients (Figure 1). Using neuropoietic coefficients allowed us to compare quantitatively six lots of SB623 cells with parental MSCs in their ability to promote the expression of rat neural progenitor marker (nestin), oligodendrocyte (CNP), and astrocyte (GFAP) markers in cocultures (Table 1).

The comparison of neuropoietic coefficients from SB623 with parental MSCs reveals a strong trend that suggests a higher neuropoietic activity of SB623 than of parental MSCs. Indeed, the majority of individual comparisons show SB623 having the higher coefficients. No differences between SB623 and MSC were observed in a few instances. In no comparison was MSC found to have a higher neuropoietic coefficient than its derivative SB623. A larger sample size, including repeated isolations from individual donors, will be required to assess the statistical significance of these observations. Our conclusion is that SB623 cells tend to show improved neuropoietic potency, compared with their parental MSCs, especially with respect to stimulating oligodendrogenesis and neural progenitor growth.

The introduction of “neuropoietic coefficients” here serves an immediate goal of comparing genetically modified MSCs with the parental MSCs with respect to one particular mechanism of action: the support of neuropoiesis. Our work could be regarded as a preliminary attempt to introduce a multiparametric measure of cell potency. This approach, however, should be further refined with regard to standardization of measurements and correlation with the *in vivo* activity. The need for such approach is dictated by the complex nature of cell therapy, and the ultimate goal is to develop a scoring system that can be used as a predictor for the *in vivo* potency.

MSCs secrete various factors with neuropoietic properties [6,25-27]. Tate *et al*. [13] identified trophic factors in conditioned media derived from MSC/SB623 pairs. We previously implicated human FGF2 as a major soluble mediator of the MSC-driven rat nestin increase in cocultures [14]. Here we showed that SB623 cells express FGF2 mRNA at higher levels (Table 2), and some SB623 lots secrete FGF2 at higher levels than do MSCs (in four of six pairs) (Additional file 4: Figure S3). Although the crucial role of FGF2 for the maintenance of neural stem cells is well known, we have shown here that small numbers of MSCs and SB623 cells can provide sufficient FGF2 quantities to drive neuropoiesis in a surrounding neural cell population. It is interesting that among three donors that had highest SB623/MSC Nes coefficient ratios, only one showed an increased FGF2 secretion in SB623 over MSCs, which supports the hypothesis that many factors determine the neuropoietic potency of a cell lot.

Other factors do contribute as well. An FGF1-blocking antibody partially reduced nestin increases driven by either the cells or the conditioned medium of MSC (Figure 2B) or of SB623 (not shown), but it had no effect on nestin increase driven by recombinant FGF2 (Figure 2B), indicating that the result is not caused by cytotoxicity. FGF1 expression in SB623 was found to be reproducibly higher than in MSC (Table 2). FGF1 is the only known FGF ligand of all four FGF receptors (FGFRs). It has been implicated in promoting neuronal maturation, rather than neurogenesis [28,29], and has demonstrated an effectiveness in an animal model of stroke by promoting neurogenesis and angiogenesis in rats [30]. FGF2 and FGF1 roles illustrate the potentially beneficial characteristic traits of MSC-based therapeutics: the redundancy and adaptability of their signals to various sets of receptors expressed by the surrounding cell population.

In SB623 cells, we observed a profound downregulation of FGF receptor FGFR2 and a mild, but reproducible, downregulation of FGFR1 expression. FGFR1 and FGFR2 are major FGF receptors expressed by cultured MSCs [31], and their downregulation may signify several things: (a) it can explain the slower growth of SB623 cells compared with MSCs [10], (b) it can be a senescence-associated trait [31]; (c) it can drastically reduce the FGF uptake by SB623 cells, thus increasing the FGF availability to the surrounding neural cells; (d) it may also represent a negative-feedback mechanism triggered by the forced expression of the exogenous NICD1 [32], a hypothesis that must be explored in subsequent studies.

SB623 cells exhibit increased levels of the BMP4 expression (Table 2). Noggin, a potent antagonist of several BMPs, including BMP2 and BMP4, as well as BMP5, BMP7, BMP13, and BMP14 (but not of BMP3, -6, –9–12, and -15) [23], significantly reduced rat GFAP levels in cocultures with SB623 (Figure 2C), indicating that noggin-inhibitable BMPs contributed to astrocyte stimulation by SB623, similar to their role in MSCs [14]. Interplay between FGF2 and BMP has been shown to control the self-renewal, dormancy, and differentiation of rat neural stem cells [33]. In our study, recombinant FGF2 and BMP4 had opposite effects on nestin-positive, as well as on GFAP-positive cells (not shown). Interestingly, the aggregated effect of the increased production of both of these “opposing” factors, as in SB623, boosted the overall neural cell growth.

MSC-produced HGF has been implicated in promoting the development of oligodendrocytes and neurons in a multiple sclerosis model [34]. HGF was not systematically affected by the SB623 manufacturing process, and its expression levels were highly variable among donors. We showed here that in cocultures, the silencing of the human HGF in SB623 cells (Figure 2D) or MSCs (not shown) partially inhibited the human-cell-mediated CNP, but not the nestin or GFAP increases. Moreover, the HGF silencing even resulted in some increases in nestin and GFAP induction, which could reflect a shift in the glia differentiation balance. These results indicated that human HGF is one of the stimuli that induce the rat oligodendrocytic marker CNP; however, other factors participate as well in the strong induction of CNP observed in SB623-driven cocultures.

We also observed that VGAT-puncta (inhibitory hemi-presynapses) were bigger and significantly more densely spaced along neurites in SB623 cocultures than in MSC cocultures, suggesting that SB623 cells are superior in promoting the GABAergic presynaptic maturation in rat embryonic neurons (Figure 3). We did not find statistically significant difference between the density of VGAT puncta in MSC cocultures and in neural cells alone, which seemingly disagreed with Mauri *et al*. [16]. Mauri *et al*. compared the effects of MSCs and astrocyte monolayers (in indirect cocultures) on both VGAT protein expression and on GABA-ergic transmission in rat embryonic hippocampal neurons and found the superior effects of MSCs. The lack of MSC effect in our study can be explained by (a) much weaker stimuli we used in our co-cultures; our plating densities were equivalent to <5% of the MSC monolayer, and by (b) differences in the detection: they quantified total immunofluorescence, whereas we counted hemisynapses.

However, at early time points, we did clearly see that in the presence of both MSCs and SB623, a diffuse VGAT staining was promoted. Mediators of VGAT stimulation were not identified here. Previously, BDNF was implicated in MSC effects on the GABAergic transmission [16]. Our preliminary experiments do not support the role of BDNF in our system. This may be explained by the very low ratios of human to rat neural cells used in this system as compared with the one described in [16].

Other factors, like FGFs, could also contribute to the enhanced effects of SB623 compared with those of MSC [18]. The enhancement of the inhibitory synapse formation might be an additional benefit of SB623 transplantations in parkinsonism, epilepsy, and movement deficits in postischemic encephalopathy, which are associated with an impairment of the GABAergic system [35].

We previously showed that SB623 cell-derived ECM supports the growth of embryonic neural cells better than does ECM derived from parental MSCs, as measured by intracellular LDH after long-term culturing [12]. Here we supplemented this observation by showing that SB623-ECM supports more robust growth of nestin-positive neural precursors than does MSC-ECM; and that the difference can be detected within 5 days (Figure 4). Importantly, in the present study, we used CellBIND microplates, which are superior to regular tissue culture plates in providing the attachment for both SB623-ECM and fragile MSC-ECM, which ensured that the difference in the neurosupportive properties of ECMs did not result from partial losses of MSC-ECM during the purification.

We hypothesize that both the observed structural robustness and the enhanced neurosupportive property of SB623-ECM, compared with those of MSC-ECM, could be a function of the same ECM molecule, tissue transglutaminase (TGM2). Indeed, four of five SB623 lots expressed more TGM2 mRNA, and the crosslinking activity of all SB623 cell lysates was higher than that of MSC lysates. We confirmed that more TGM2 is present in SB623-derived ECM than in MSC-derived ECM (Figure 5). When TGM2 expression was efficiently silenced (by using siRNA), the ECM produced by SB623 cells with silenced TGM2 supported less nestin-positive cell growth than did the control ECM (Figure 5D). In a coculture setting, however, neuropoietic properties of SB623 were not affected by the TGM2 silencing (Figure 5E). These facts suggested that TGM2 mediated its effect on neural precursors when it was ECM-associated, rather than cell surface-associated or intracellular. Also, the silencing of TGM2 was fairly specific, and the transfectants did not exhibit global health issues compared with the control transfectants (Additional file 7: Figure S6).

These observations implicate TGM2 in the improved neuropoietic activity of SB623 ECM; however, the nature of its contribution remains unclear. We speculate that the ECM-associated TGM2 could promote neural cell growth in ECM-based cocultures by means of enzymatic crosslinkng activity and nonenzymatic adapter/scaffolding function. Indeed, the cell-surface TGM2 nonenzymatically promotes a rapid assembly of fibronectin and remains associated with fibronectin fibrils [36]. This event initiates the assembly and crosslinking of other components of ECM including fibrin, fibronectin, collagen, vitronectin, and osteopontin [37]. This way, TGM2 increases ECM stability and the rigidity of crosslinked fibronectin and collagen fibrils. Several reports emphasized that substrate stiffness *per se* can govern the fate of neural stem cells [38-40]. They showed that neural cell proliferation favors soft substrates, neuronal differentiation even softer ones, whereas oligodendrogenesis needed stiffer substrates. This seeming contradiction, that presumably stiffer SB623-ECM exhibits superior neuropoietic properties to those of MSC-ECM, could be resolved if we consider the other consequences of TGM2-crosslinking activity: unmasking cryptic binding sites within ECM molecules for other ECM components and cell-surface receptors, such as integrins [41,42]. Besides, TGM2 deposited in ECM binds with high affinity to heparin sulfate moieties of syndecan-4 [43]. Both integrins and syndecan-4 are essential for the adhesion, survival, and proliferation of nestin-positive neural cells and are colocalized with neural stem cells in brain development [19,44,45]. Thus, neural precursors and differentiating cells could benefit from an increased activity and enhanced incorporation of TGM2 into ECM as observed in SB623 cells in numerous ways. Moreover, the higher expression of TGM2 can also be beneficial for the survival of transplanted SB623 themselves. Indeed, TGM2-overexpressing MSC showed an improved survival after transplantation when used in a myocardial injury model [24]. Interestingly, another crosslinking enzyme secreted to the medium, quiescin sulfhydryl oxidase 1, was recently reported to participate in laminin incorporation to ECM and thus be required for cell adhesion and migration [46].

## Conclusions

The data presented here demonstrate that MSC-derived cells, SB623, which are in development for intracranial transplantations to treat stable stroke, are typically more potent than their parental MSCs in stimulating the growth and differentiation of neural precursors. This increased neuropoietic potency of SB623 observed in cocultures with rat embryonic cortex cells can be attributed to the increased expression of FGF1, FGF2, and BMPs in SB623 as compared to MSCs. The presented data also indicate that the ECM produced by SB623 promotes a more efficient neural precursor growth than does the ECM produced by MSC, which can be at least partly explained by higher activity and expression levels of an ECM crosslinking enzyme, TGM2, observed in SB623 compared with parental MSCs. The enhanced neuropoietic potential of SB623 could be beneficial in a neurodegenerative application, where the stimulation and support of endogenous neural precursors constitute one of the proposed mechanisms of action of cell therapeutic transplantations. Further studies are required to determine how genetic manipulation and other manufacturing steps contribute to the creation of this new phenotype of SB623.

## Abbreviations

bFM1 and bFM2: clones of neutralizing and nonneutralizing anti-FGF2 mouse antibodies correspondingly; BMP: bone morphogenetic protein; BSA: bovine serum albumin; CM: conditioned medium; CNP: 2′,3′-cyclic-nucleotide 3′-phosphodiesterase; ECM: extracellular matrix; EGF: epidermal growth factor; FBS: fetal bovine serum; FGF: fibroblast growth factor; FGFR: fibroblast growth factor receptor; GABA: γ-aminobutyric acid; GAP: glyceraldehyde 3-phosphate dehydrogenase; GDI: rho GDP dissociation inhibitor alpha; GFAP: glial fibrillary acidic protein; GLX: GlutaMAX; HB-EGF: heparin binding EGF-like growth factor; HGF: hepatocyte growth factor; LAMA4: lLaminin: alpha 4; LDH: lactate dehydrogenase; MSC: mesenchymal stromal cells; NB: neurobasal medium; Nes: nestin; NICD1: Notch1 intracellular domain; PBS: phosphate-buffered saline; PLL: Poly-L-lysine; PS: penicillin and streptomycin; qRT-PCR: quantitative reverse-transcription PCR; siRNA: small interfering RNA; TGM2: tissue transglutaminase; VGAT: vesicular GABA transporter; VGLUT: Vesicular glutamate transporter; αMEM: α-minimal essential medium.

## Competing interests

IA, MM, and CCC are employees of SanBio, Inc. The authors declare that they have no competing interests.

## Authors’ contributions

IA designed the study, performed experiments, analyzed and interpreted data, and wrote the manuscript. BT performed TGM2-related experiments, analyzed and interpreted the data, and participated in drafting the manuscript. MM directed the production and characterization of MSC and SB623, participated in data interpretation and the manuscript drafting and editing. CCC supervised the research, participated in data analysis and interpretation, critically revised and edited the manuscript. All authors read and approved the final manuscript.

## Supplementary Material

Additional file 1: Figure S1 Immunodetection of neurons in cocultures. Cocultures described in Figure 1 were stained for MAP2, and counterstained with DAPI on day 5 and day 9 of culturing. No difference between MSC and SB623 cocultures was detected in either numbers of mature neurons or their dendrite outgrowth at these time points. Click here for file

Additional file 2: Figure S2 Quantification of immunostaining of nestin, GFAP, and CNP in cocultures. Neural cells were cultured either alone or with MSC or SB623 (Donor D) at rat to human cell ratio 20:1 Cultures were immunostained for either nestin or GFAP on day 5 or CNP on day 12; and counterstained with DAPI. Immunofluorescence signal from each neuromarker was quantified by using ImageJ and results expressed as immunofluorescence per positive cell. Error bars represent the standard deviation between four fields, from duplicated cultures. *P < 0.05. Click here for file

Additional file 3: Table S1 Ratios of mRNA expression by donor, SB623/MSC. Click here for file

Additional file 4: Figure S3 FGF2 secretion by MSC and SB623, ELISA. Neurobasal medium was conditioned by confluent MSC or SB623 layers for 1 day. FGF2 ELISA was eventually performed on these aliquots by using the R&D System Duo set of antibodies, MaxiSorp plates (Nunc), and recombinant FGF2 from Peprotech for standard curve. After the removal of the conditioned medium, cells were lysed in 2% Triton, and LDH in lysates was quantified as a surrogate for cell-number determination. The graph shows FGF2 concentrations normalized to LDH. All samples in this graph were analyzed in the same experiment. Click here for file

Additional file 5: Figure S4 Comparison of vital functions in HGF and control siRNA transfectants. **(A)** MSCs were transfected with either HGF siRNA (siHGF) or control siRNA (siControl) or not transfected at all; next day, they were replated and grown for additional 5 days (6 days after transfection) in six well plates, then harvested and counted by using trypan blue to determine the total numbers of harvested cells and their viability. The number of harvested transfectants was expressed as percentage of harvested not-transfected cells. Error bars represent standard deviations in duplicated samples. **(B)** MSCs transfected as above were replated in 96-well plates in quadruplicates and grown for a total of 3 and 6 days after transfection. On indicated days cells were lysed by using SideStep Lysis buffer and replicas were combined pair-wise, resulting in biologic duplicates. One-step qRT-PCR was performed on water-diluted samples using preoptimized Taqman assays; and results were normalized to GAP. The expression level of each gene in Control siRNA transfectants (siControl) was set on 1, and the level in HGF siRNA transfectants was expressed correspondingly. Error bars represent standard deviations between biologic duplicates. Click here for file

Additional file 6: Figure S5 TGM2 in ECM in two pairs of MSC/SB623. Two duplicated gels were electrophoresed; and one was transferred for immunoblotting with TGM2 antibody, whereas another one was stained for protein. Labels on irrelevant samples are omitted. TGM2 antibody detected a single band in ECM, around ~80 kDa. Both blot and gel were analyzed densitometrically. Click here for file

Additional file 7: Figure S6 Comparison of vital functions in TGM2 and control siRNA transfectants. **(A)** SB623 were transfected with either TGM2 siRNA (siTGM2) or control siRNA (siControl) or not transfected at all; on the next day, cells were replated and grown for additional 5 days (6 days after transfection) in six-well plates, then harvested and counted by using trypan blue to determine the total number of harvested cells and their viability. The number of harvested transfectants was expressed as a percentage of harvested not-transfected cells. Error bars represent standard deviations in duplicated samples. **(B)** SB623 transfected as described were replated in 96-well plates in quadruplicates and grown for total 3 and 6 days after transfection. On indicated days cells were lysed by using SideStep Lysis buffer, and replicas were combined pair-wise resulting in biologic duplicates. One-step qRT-PCR was performed on water-diluted samples by using preoptimized Taqman assays; and results were normalized to GAP. The expression level of each gene in Control siRNA transfectants (siControl) was set on 1, and the level in TGM2 siRNA transfectants was expressed correspondingly. Error bars represent standard deviations between biologic duplicates. Click here for file
