# Supplementary figures and images for: Comparison of the neuropoietic activity of gene-modified versus parental mesenchymal stromal cells and the identification of soluble and extracellular matrix-related neuropoietic mediators
Source: Stem Cell Res Ther. 2014 Feb 26;5(1):29. doi: 10.1186/scrt418 (PMC4055059; doi:10.1186/scrt418)

N

N+MSC

N+SB623

5 days

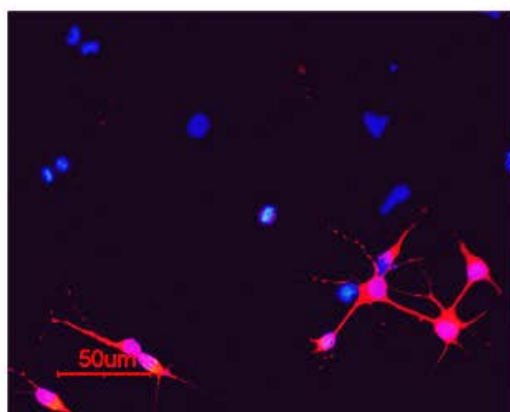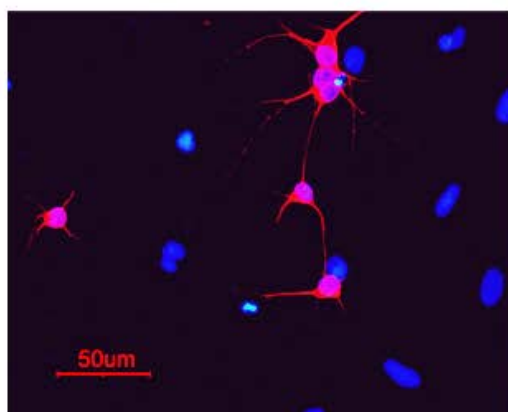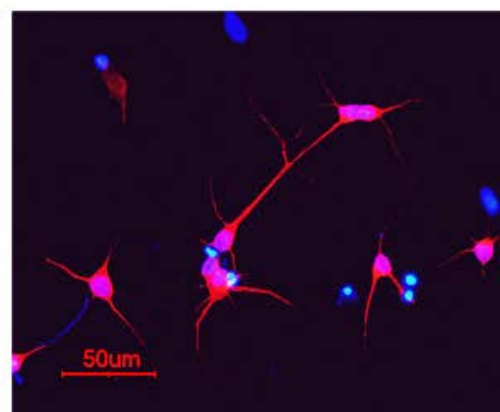

9 days

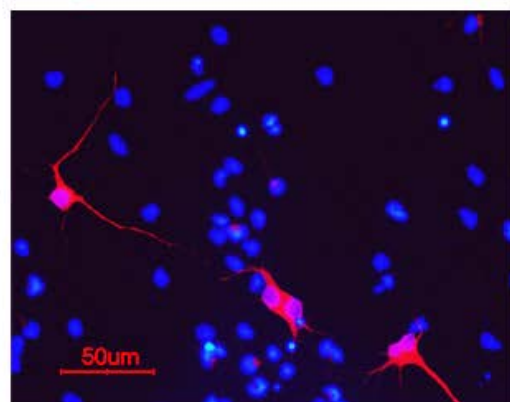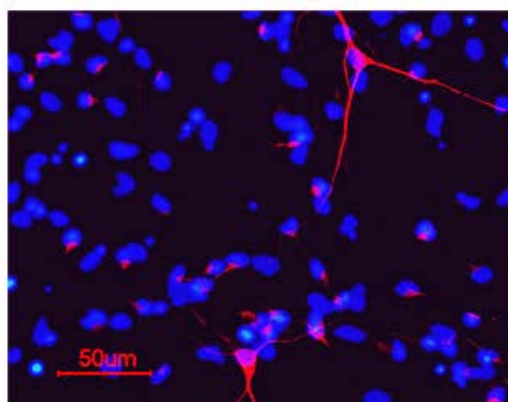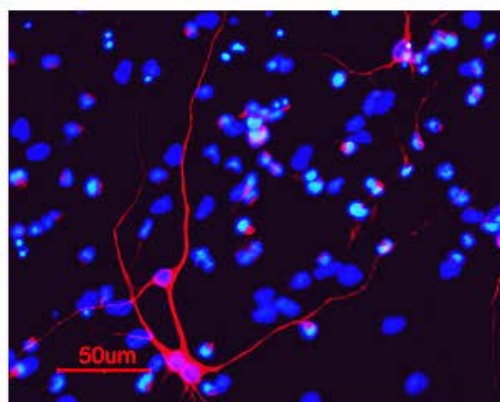

Supplement: Additional file 1: Figure S1 — Immunodetection of neurons in cocultures. Cocultures described in Figure 1 were stained for MAP2, and counterstained with DAPI on day 5 and day 9 of culturing. No difference between MSC and SB623 cocultures was detected in either numbers of mature neurons or their dendrite outgrowth at these time points. [file scrt418-S1.pdf]

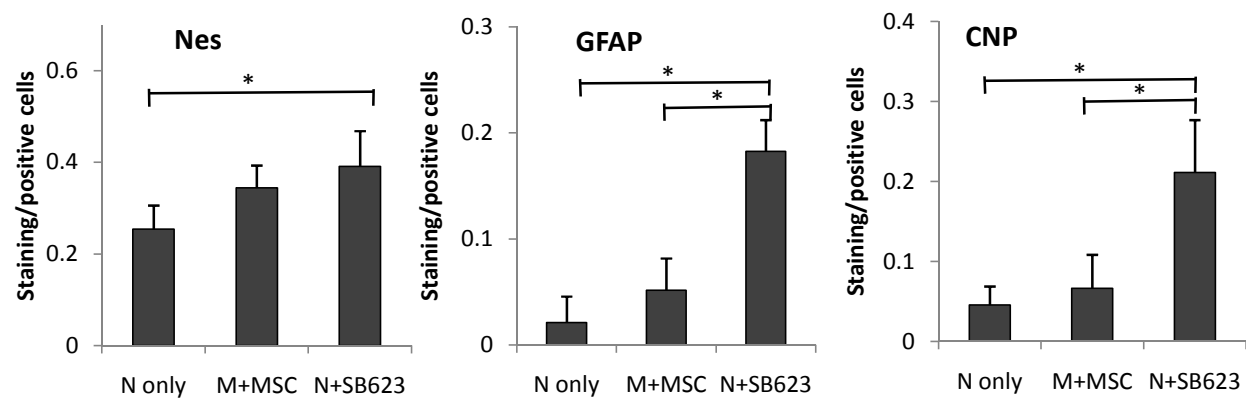

Supplement: Additional file 2: Figure S2 — Quantification of immunostaining of nestin, GFAP, and CNP in cocultures. Neural cells were cultured either alone or with MSC or SB623 (Donor D) at rat to human cell ratio 20:1 Cultures were immunostained for either nestin or GFAP on day 5 or CNP on day 12; and counterstained with DAPI. Immunofluorescence signal from each neuromarker was quantified by using ImageJ and results expressed as immunofluorescence per positive cell. Error bars represent the standard deviation between four fields, from duplicated cultures. *P < 0.05. [file scrt418-S2.pdf]

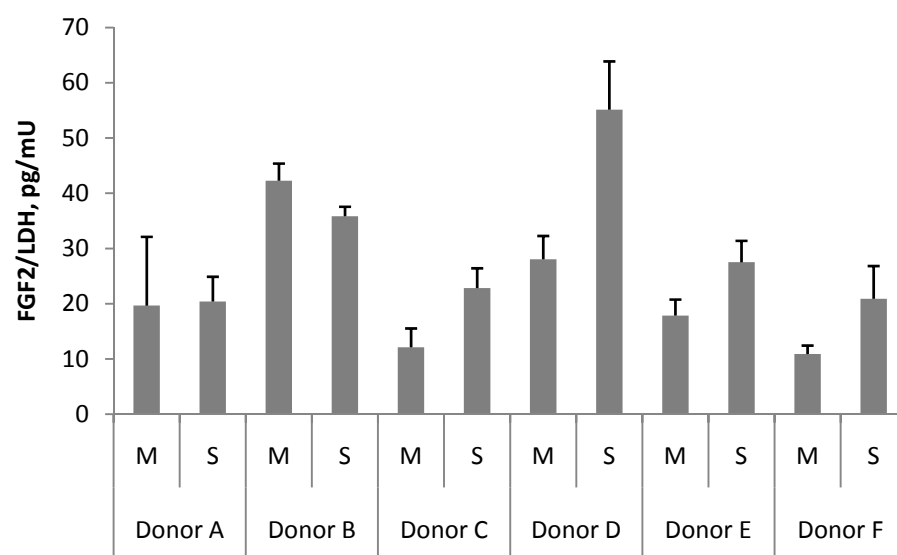

Supplement: Additional file 4: Figure S3 — FGF2 secretion by MSC and SB623, ELISA. Neurobasal medium was conditioned by confluent MSC or SB623 layers for 1 day. FGF2 ELISA was eventually performed on these aliquots by using the R&D System Duo set of antibodies, MaxiSorp plates (Nunc), and recombinant FGF2 from Peprotech for standard curve. After the removal of the conditioned medium, cells were lysed in 2% Triton, and LDH in lysates was quantified as a surrogate for cell-number determination. The graph shows FGF2 concentrations normalized to LDH. All samples in this graph were analyzed in the same experiment. [file scrt418-S4.pdf]

**A**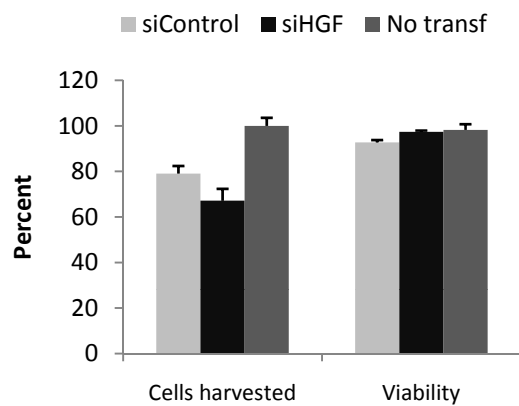**B**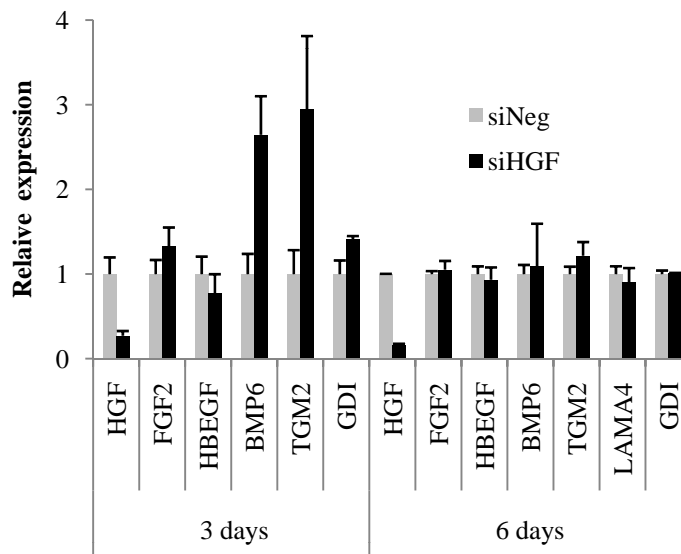

Supplement: Additional file 5: Figure S4 — Comparison of vital functions in HGF and control siRNA transfectants. (A) MSCs were transfected with either HGF siRNA (siHGF) or control siRNA (siControl) or not transfected at all; next day, they were replated and grown for additional 5 days (6 days after transfection) in six well plates, then harvested and counted by using trypan blue to determine the total numbers of harvested cells and their viability. The number of harvested transfectants was expressed as percentage of harvested not-transfected cells. Error bars represent standard deviations in duplicated samples. (B) MSCs transfected as above were replated in 96-well plates in quadruplicates and grown for a total of 3 and 6 days after transfection. On indicated days cells were lysed by using SideStep Lysis buffer and replicas were combined pair-wise, resulting in biologic duplicates. One-step qRT-PCR was performed on water-diluted samples using preoptimized Taqman assays; and results were normalized to GAP. The expression level of each gene in Control siRNA transfectants (siControl) was set on 1, and the level in HGF siRNA transfectants was expressed correspondingly. Error bars represent standard deviations between biologic duplicates. [file scrt418-S5.pdf]

Markers  
 MSC-C-ECM  
 SB623-C-ECM  
 -  
 -  
 -  
 -  
 -  
 MSC-G-ECM  
 SB623-G-ECM  
 Whole cell lysate

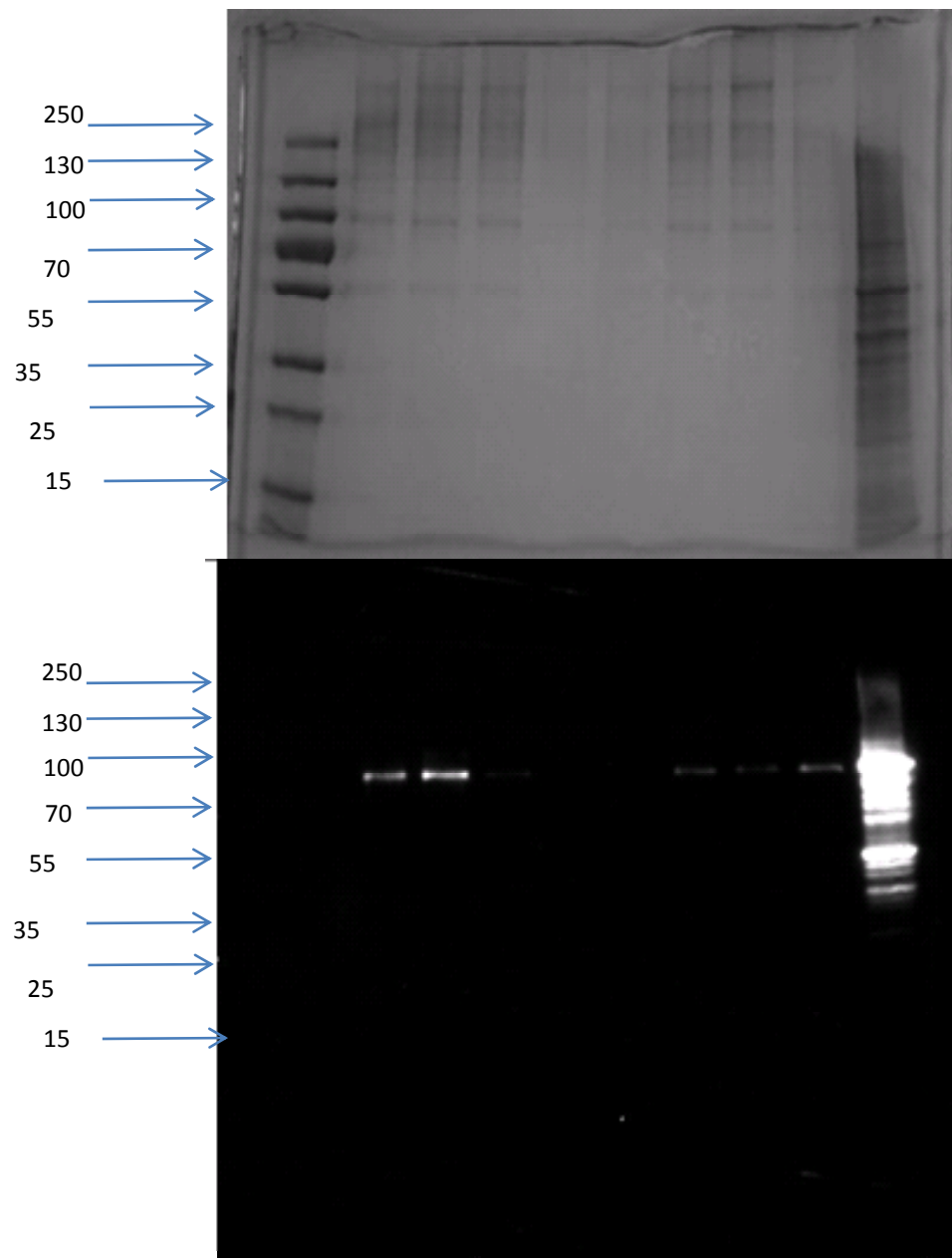

Supplement: Additional file 6: Figure S5 — TGM2 in ECM in two pairs of MSC/SB623. Two duplicated gels were electrophoresed; and one was transferred for immunoblotting with TGM2 antibody, whereas another one was stained for protein. Labels on irrelevant samples are omitted. TGM2 antibody detected a single band in ECM, around ~80 kDa. Both blot and gel were analyzed densitometrically. [file scrt418-S6.pdf]

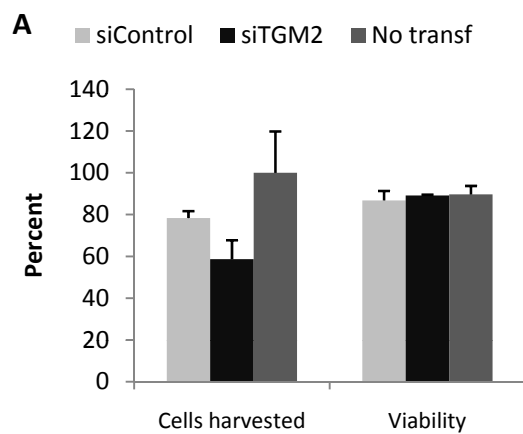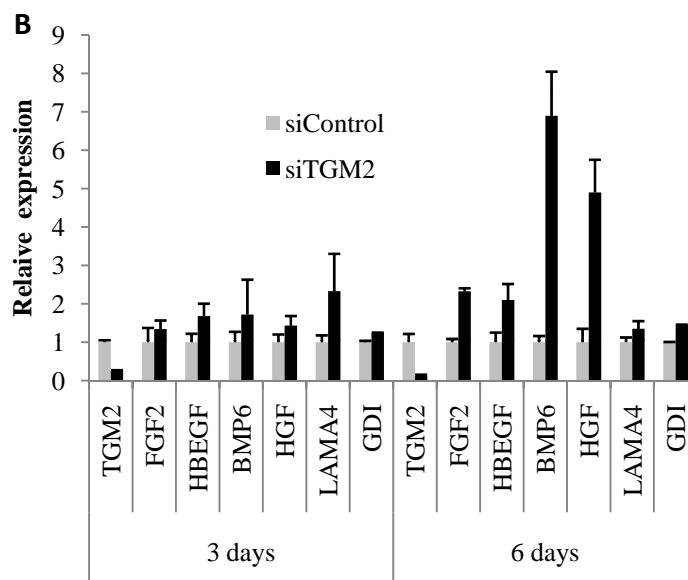

Supplement: Additional file 7: Figure S6 — Comparison of vital functions in TGM2 and control siRNA transfectants. (A) SB623 were transfected with either TGM2 siRNA (siTGM2) or control siRNA (siControl) or not transfected at all; on the next day, cells were replated and grown for additional 5 days (6 days after transfection) in six-well plates, then harvested and counted by using trypan blue to determine the total number of harvested cells and their viability. The number of harvested transfectants was expressed as a percentage of harvested not-transfected cells. Error bars represent standard deviations in duplicated samples. (B) SB623 transfected as described were replated in 96-well plates in quadruplicates and grown for total 3 and 6 days after transfection. On indicated days cells were lysed by using SideStep Lysis buffer, and replicas were combined pair-wise resulting in biologic duplicates. One-step qRT-PCR was performed on water-diluted samples by using preoptimized Taqman assays; and results were normalized to GAP. The expression level of each gene in Control siRNA transfectants (siControl) was set on 1, and the level in TGM2 siRNA transfectants was expressed correspondingly. Error bars represent standard deviations between biologic duplicates. [file scrt418-S7.pdf]
