# Supplementary material for: Comparison of the neuropoietic activity of gene-modified versus parental mesenchymal stromal cells and the identification of soluble and extracellular matrix-related neuropoietic mediators
Source: Stem Cell Res Ther. 2014 Feb 26;5(1):29. doi: 10.1186/scrt418 (PMC4055059; doi:10.1186/scrt418)
Supplement: Additional file 3: Table S1 — Ratios of mRNA expression by donor, SB623/MSC. [file scrt418-S3.pdf]

**Additional table 1. Ratios of mRNA expression by donor, SB623/MSC.**

| Donor ID | FGF1 | FGF2 | FGFR1 | FGFR2 | BMP2 [&] | BMP4 | BMP6 | HB-EGF | EGF | HGF |
|---|---|---|---|---|---|---|---|---|---|---|
| **A** | 1.31 | 1.75 | 0.54 | 0.27 | | 6.73 | ND | 0.86 | 2.06 | 0.26 |
| **B** | 1.63 | 1.41 | 0.79 | 0.27 | | 1.99 | 0.59 | 0.86 | 1.92 | 0.23 |
| **C** | 1.20 | 1.79 | 0.69 | 0.32 | | 2.25 | 2.63 | 0.60 | 0.65 | 0.69 |
| **D** | 1.33 | 1.31 | 0.82 | 0.27 | | 7.31 | 0.77 | 0.62 | 1.41 | 0.41 |
| **E** | 1.71 | 2.70 | 0.73 | 0.17 | | 4.36 | 3.12 | 1.63 | 2.70 | 0.87 |
| **F** | 0.95 | 1.23 | 0.95 | 0.36 | | 2.25 | 1.91 | 0.87 | 0.55 | 2.37 |
| **H** | 1.09 | 1.72 | 0.83 | 0.15 | | 1.86 | ND | 0.45 | 0.91 | 0.70 |
| **G** | 1.57 | 2.02 | 0.64 | 0.13 | | 1.92 | 0.56 | 1.71 | 1.69 | 0.12 |

[&] Expression levels too low
